# Extended *Q*-range small-angle neutron scattering to understand the morphology of proton-exchange membranes: the case of the functionalized syndiotactic-polystyrene model system

**DOI:** 10.1107/S1600576723005496

**Published:** 2023-07-25

**Authors:** Maria-Maddalena Schiavone, David Hermann Lamparelli, Christophe Daniel, Manuchar Golla, Yue Zhao, Hiroki Iwase, Hiroshi Arima-Osonoi, Shin-ichi Takata, Laszlo Szentmiklosi, Boglarka Maroti, Jürgen Allgaier, Aurel Radulescu

**Affiliations:** a Jülich Centre for Neutron Science (JCNS) at Heinz Maier-Leibnitz Zentrum (MLZ), Forschungszentrum Jülich GmbH, Lichtenbergstrasse 1, Garching, 85747, Germany; b Dipartimento di Chimica e Biologia ‘Adolfo Zambelli’, Università di Salerno, Fisciano, I-84084, Italy; c Takasaki Advanced Radiation Institute, National Institutes for Quantum Science and Technology, Takasaki, 370-1292, Japan; d Neutron Science and Technology Centre, Comprehensive Research Organization for Science and Society CROSS, Tokai, 319-1106, Japan; e Materials and Life Science Division, Japan Proton Accelerator Complex J-PARC, Tokai, 319-1195, Japan; f Institute for Energy Security and Environmental Safety, Centre for Energy Research, Budapest, 1121, Hungary; g Jülich Centre for Neutron Science (JCNS), Forschungszentrum Jülich GmbH, Jülich, 52425, Germany; Argonne National Laboratory, USA

**Keywords:** pinhole SANS, small-angle neutron scattering, TOF SANS, time of flight, WANS, wide-angle neutron scattering, ion-conducting polymer membranes

## Abstract

Semi-crystalline polymers exhibit microphase separation into crystalline and amorphous domains characterized by multiple structural levels with sizes ranging from ångströms to hundreds of nanometres. The combination of small-angle (SANS) and wide-angle (WANS) neutron scattering on the same beamline enables reliable *in situ* characterization of such materials under application-relevant conditions, with the unique advantage of contrast variation by controlled labelling, allowing the structure of such multi-component systems to be resolved in detail. This paper reports a structural analysis performed on deuterated polymer membranes based on syndiotactic polystyrene (sPS) using an extended *Q*-range SANS and WANS combination, always with the same neutron scattering instrument, either a pinhole SANS diffractometer installed at a research reactor or a ‘small- and wide-angle’ time-of-flight diffractometer installed at a neutron spallation source. sPS is a semi-crystalline material that becomes hydrophilic and proton conducting when suitable functionalization is achieved by thin film sulfonation, and can form various co-crystalline complexes (clathrates) with small organic molecules stored in the crystalline phase as guests in the vacancies between the polymer helices. Therefore, this material is interesting not only for its conducting properties but also for its versatility as a model system to evaluate the usefulness of extended *Q*-range neutron scattering in such studies. Variation of neutron contrast was achieved in the amorphous hydrophilic phase by using H_2_O or D_2_O to hydrate the membranes and in the crystalline phase by loading the clathrates with deuterated or protonated guest molecules. The experimental approach, the advantages and limitations of the two types of instrumentation used in such analyses, and the main results obtained with respect to the structural characterization of sulfonated sPS membranes under different hydration and temperature conditions are reported, and the potential of this method for similar structural studies on other semi-crystalline polymeric materials is discussed.

## Abstract 2

Semi-crystalline polymers present a hierarchical organization of structural levels from ångströms to hundreds of nanometres. By combining small- and wide-angle neutron scattering at the same instrument, such complex morphologies can be resolved under application of relevant humidity and temperature conditions.

## 1. Introduction

Syndiotactic polystyrene (sPS) is a relatively new material (Ishihara *et al.*, 1986 ▸) that shows a very complex polymorphic behaviour, including five different crystalline forms in which the chains adopt either a planar zigzag (α and β form) or a TTGG helical (γ, δ and ε form) conformation (Guerra *et al.*, 1990 ▸; Chatani *et al.*, 1992 ▸; Gowd *et al.*, 2009 ▸). Moreover, sPS is able to form different kinds of co-crystalline (clathrate) phases with a large number of guest molecules which can be incorporated in the cavities between the helices of the crystalline region of the δ and ε forms (Tarallo *et al.*, 2010*a* ▸,*b* ▸, 2011 ▸). The clathrate forms are not only interesting for applications, when active guests can be incorporated in the sPS films, leading to advanced materials for optical and magnetic applications (Giordano *et al.*, 2005 ▸; Stegmaier *et al.*, 2005 ▸; Daniel *et al.*, 2009 ▸; Rizzo *et al.*, 2010 ▸), but may offer a unique advantage in the case when neutron scattering methods are used for the investigation of the microstructure and microdynamics of such materials; the neutron scattering length density (SLD) of the crystalline regions can be varied by loading D and H isotopologues of the guest molecules in the crystalline cages of the δ form and channels of the ε form while working with deuterated sPS matrix. This advantage has already been demonstrated in some previous small-angle neutron scattering (SANS) studies (Kaneko *et al.*, 2013 ▸, 2014 ▸). Moreover, using the selectivity of the δ form, a homogeneous functionalization by sulfonation of sPS films as thick as 200 µm can be achieved (Borriello *et al.*, 2009 ▸); using a bulky sulfonating agent (the lauroyl sulfate), which is unable to enter the clathrate crystalline phase, the sulfonation can occur only in the amorphous phase, while the use of chloro­form as solvent, which is able to easily penetrate the clathrate crystalline phase, makes the sulfonation of the amorphous phase rapid and uniform throughout the whole film thickness. In contrast to the uniform sulfonation of films containing the δ clathrates, films with dense crystalline phases (*e.g.* the β phase) are not permeable to the solvent and are sulfonated only very slowly and mainly at the film surface.

Sulfonated syndiotactic polystyrene (s-sPS) is hydro­philic and shows a high proton conductivity comparable to that of Nafion (Gianluca *et al.*, 2011 ▸; Schiavone *et al.*, 2022 ▸), which is the benchmark in proton-exchange membrane fuel cell technology (Kusoglu & Weber, 2017 ▸). Thus, s-sPS membranes may become suitable for ion-conducting applications since they are characterized by a nanoscale phase separation into hydro­philic domains and hydro­phobic regions. This combination enables a high ion conductivity and provides a good chemical and thermomechanical stability. The ion conduction in such membranes depends on water and is governed by the structural and dynamical behaviour of water at the nano- and mesoscale. To understand the ion-transport properties in different application-relevant conditions, one should first of all understand the morphology of hydrated domains at different length scales as a function of hydration level, which depends on the relative humidity (RH) and water uptake capacity, and temperature (*T*). Typically, such membranes swell upon hydration, while degradation may occur when operating in harsh chemical environments, like in the presence of free radicals of hydroxyl and hydro­peroxyl that are formed in fuel cells, for example. Both effects have a negative impact on membrane crystallinity and mechanical stability. Therefore, to understand the structural properties of such membranes, it would be of great advantage to be able to analyse the mechanical and chemical stability in parallel with the characterization of the hydrated morphology, which means that one should be able to observe the crystalline structure on the short length scale simultaneously with the large-scale morphology. Characterization over a broad length scale, between ångströms and hundreds of nanometres, usually requires a combination of different experimental methods in structural analysis. Scattering methods with X-rays or neutrons in the small- (SAS) and wide-angle (WAS) regimes have long been used for such experimental investigations (Kanaya *et al.*, 1998 ▸; Hashimoto *et al.*, 1995 ▸). WAS provides information about the crystalline structure whereas SAS delivers complex information about the higher organizational level of molecules such as lamellae, interlamellar amorphous and bulk amorphous morphology, lamellar aggregates, and the overall membrane morphology. Unlike X-rays, neutrons ‘see’ light atoms in the presence of heavier ones. In particular, the strong difference in neutron cross section between the hydrogen isotopes protium (^1^H) and deuterium (^2^H) provides a unique advantage for neutrons in the study of hydro­carbon materials such as synthetic or natural polymers, combining SANS and wide-angle neutron scattering (WANS) techniques with the contrast matching/variation method (Kanaya *et al.*, 2007 ▸). Due to the complexity of the experimental approach, which involves *in situ* variation of RH and *T* or chemical treatment of the samples, a fully reliable analysis of this type should preferably be performed at the same diffractometer over an extended *Q* range that allows simultaneous acquisition of SANS and WANS patterns.

In previous work (Schiavone *et al.*, 2018 ▸, 2019 ▸, 2020 ▸, 2022 ▸) we reported a detailed microstructural characterization of ion-conducting membranes based on s-sPS with the crystalline δ form using SANS with an extended *Q* range (up to *Q*_max_ = 1.0 Å^−1^), where, in addition to the SANS features, the 010 crystalline reflection of the sPS lattice (Rizzo *et al.*, 2002 ▸) was always observed in the scattering patterns detected with a wavelength resolution Δλ/λ = 10%, typical of pinhole SANS instruments. Samples characterized by different degrees of sulfonation (SD) and crystallinity, as confirmed by prompt-gamma neutron activation analysis (PGAA), Fourier-transform infrared spectroscopy (FTIR) and X-ray diffraction (XRD), were subjected to *in situ* variation of RH and *T*. The 010 reflection always occurred in neutron scattering patterns at *Q*_010_ = 0.56 Å^−1^, indicating that the crystallinity of the membrane was maintained to a large extent during the sample treatment and characterization. The extended WAS patterns in XRD experiments contain the crystalline reflections that are used to identify the crystalline form and the state of the guest loading in the sPS clathrates at a first inspection of such experimental data. At a high content of guest molecules stored in the cavities between the sPS helices (14 wt%; De Rosa *et al.*, 1997 ▸), two reflections were observed in the XRD patterns of the δ form, 010 and the 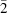 10 at scattering angles of 2θ ≈ 8 and 10°, respectively. These two peaks behave in the opposite way as the amount of guest decreases in the clathrates; the reflection at 2θ ≈ 10° decreases until it almost disappears in the case of the empty clathrate form, while the intensity of the 2θ ≈ 8° reflection increases. Moreover, annealing of sPS samples at high temperature causes a transition from the δ form to the β form, which is thermodynamically more stable and more suitable for energy applications (Gianluca *et al.*, 2011 ▸). Therefore, it would be highly desirable to record the WANS patterns at higher values than *Q*_max_ = 1.0 Å^−1^ achieved in our previous experiments and at a higher instrumental resolution to capture in detail the scattering features of the crystalline structure simultaneously with recording the scattering from the large-scale morphologies.

sPS is a system characterized by a large unit cell in the δ and ε forms (De Rosa *et al.*, 1997 ▸), and thus provides crystalline reflections at lower *Q* values than those normally involved in scattering experiments on other semi-crystalline polymers. Therefore, this system is perfectly suited to optimize the experimental conditions and prove the power of the combination of SANS and WANS in the characterization of semi-crystalline ion-conducting polymer membranes with *in situ* variation of external stimuli on the sample. Note also that the combination of SANS and WANS has already been tested in the crystallization of semi-crystalline polymers from melts, for polymeric systems with crystalline domains characterized by a smaller unit cell, such as deuterated polyethylene (Kanaya *et al.*, 2007 ▸) or poly(ε-caprolactone) (Mitchell *et al.*, 2015 ▸), where the reflections of the crystalline structure occur at higher *Q* values than in the sPS system.

In this work we report the advantages offered by the experimental characterization of semi-crystalline s-sPS ionic membranes in a moderate hydration regime at different *T* by using the extended *Q*-range SANS/WANS combination up to *Q*_max_ = 5.0 Å^−1^ at the time-of-flight (TOF) ‘small- and wide-angle’ diffractometer BL-15 TAIKAN, installed at the spallation source of the Material and Life Science Experimental Facility of the Japan Proton Accelerator Research Facility (J-PARC), Tokai, Japan (Takata *et al.*, 2015 ▸). A parallel with the SANS investigation using the pinhole SANS diffractometer KWS-2 of Jülich Centre for Neutron Science at Forschungs-Neutronenquelle Heinz Maier-Leibnitz (FRM II), Germany, is discussed. Hydrated morphologies and long-range crystalline correlations were characterized through the analysis of the scattering patterns from membranes in the SANS regime while the crystalline structure was revealed by the WANS data. As-cast and uniaxially deformed films in the partially free water regimes were used in this experiment. We show here how adjusting the experimental conditions in terms of the instrument resolution and the sample composition via the variation of SLD in the crystalline region can improve the quality of the results and how the structural characterization of such complex morphologies can be done in an optimal way.

## 2. Experiment

### 2.1. Sample preparation

Deuterated syndiotactic polystyrene (d8-sPS) was synthesized using d8-styrene monomers (purchased from Sigma Aldrich, isotopic purity 98 at.% D), which were purified by distillation under reduced pressure over CaH_2_, and a homogeneous catalytic system composed of penta­methyl­cyclo­pentadienyltitanium trichloride (purchased from Strem Chemicals) and methyl­alumoxane (purchased from Chemtura) in toluene (purchased from Sigma Aldrich); the mixture was refluxed for 48 h over metallic sodium and distilled under a nitro­gen atmosphere. The polymerization reaction was carried out in a 250 ml glass reactor equipped with a magnetic stirrer which was loaded with chemicals under a nitro­gen atmosphere and thermostated at 313 K in an oil bath. The polymerization was stopped after 12 h by injecting acidified methanol. The polymer was recovered by filtration, washed with fresh methanol and dried in vacuum at 333 K. The obtained polymer possesses excellent syndiotacticity (fraction of the *rrrrrr* heptad of 95%), as determined by ^13^C NMR spectroscopy. Molecular weights (*M*_w_ and *M*_n_) and polydispersity (*M*_w_/*M*_n_) were determined by high-temperature gel permeation chromatography (GPC) at 413 K. The deuterated syndiotactic polystyrene used in this work presents a weight-average molar mass *M*_w_ of 1.37 × 10^6^ g mol^−1^ with a dispersity index *M*_w_/*M*_n_ = 2.09.

Oriented sPS deuterated films were obtained by stretching amorphous films prepared by melt pressing at 553 K followed by quenching at 273 K in an ice/water mixture. The amorphous film stretching was performed with an INSTRON 4301 dynamometer at 383 K at a speed of 10 mm min^−1^ up to a draw ratio of 3.2. Then, axially oriented films with the δ form were obtained by exposure to toluene vapours.

Un-oriented δ-form clathrate samples of deuterated sPS with guest molecules incorporated by the crystalline regions were obtained by casting a polymer–toluene solution onto a glass substrate to form a film. The solution was prepared at 2 wt% polymer and then heated in sealed glass flasks to about 413 K with stirring for 2 h until complete polymer dissolution. The solution was subsequently poured into a Petri dish to obtain the cast film directly in the δ co-crystalline form with protonated toluene as guest molecule. The thickness of the cast and drawn films was about 50–100 µm. The guest mol­ecules incorporated in the crystalline regions of the sPS films were exchanged between protonated and deuterated toluene (H-Tol or D-Tol) by using the guest-exchange mechanism (Kaneko *et al.*, 2013 ▸, 2014 ▸) through immersion of the films in appropriate solvents for several hours.

Films with variable degrees of sulfonation were produced via the solid-state sulfonation procedure involving the lauroyl sulfate solution in CDCl_3_, which allowed a uniform sulfonation of the phenyl rings of the amorphous phase and preserved the crystalline δ form (Borriello *et al.*, 2009 ▸). Film crystallinity was controlled before and after sulfonation by XRD in the range of 2θ between 5 and 30° by means of a Bruker second-generation D2 Phaser X-ray powder diffractometer (Cu source). Orientation or the isotropic character of films was controlled using a Rigaku single-crystal diffractometer (Cu and Mo sources) equipped with a HyPix-Arc 150° curved hybrid photon counting X-ray detector. The crystallinity of the polymer films was evaluated using the data analysis software at the X-ray powder diffractometer. The effect of the sulfonation reaction and guest-exchange procedure was checked by FTIR using a JASCO VIR-200 spectrometer in a wavenumber range of 400–4000 cm^−1^. The SD of s-sPS membranes expressed as S atoms/styrene units × 100 mol% was checked at the PGAA instrument at the Budapest Neutron Centre (BNC, Budapest, Hungary). Full descriptions of the experimental method and data interpretation are reported by Révay (2009 ▸).

The guest loading in the crystalline region was quantified by thermogravimetric analysis (TGA) using a NETZSCH TG 209 F1 Libra instrument (NETZSCH-Gerätebau GmbH, Selb, Germany) in the temperature range 303–723 K at a heating rate of 5 K min^−1^ with a nitro­gen flow of 60 ml min^−1^.

The ionic exchange capacity (IEC, expressed in meq g^−1^) was evaluated following a procedure of sample treatment in 1.2*M* hydro­chloric acid for 12 h, to ensure protonation of all the sulfonic acid groups, followed by a subsequent soaking in 20 ml of 0.1*M* NaCl for 2 days, to provide the exchange of ions, and the measurement of the pH of the proton-exchanged NaCl solution. The details of the IEC determination procedure can be found elsewhere (Schiavone *et al.*, 2018 ▸). The water uptake (WU) capacity of the membranes was determined by following the preparation procedure described by Schiavone *et al.* (2018 ▸), and expressed as the percentage increase in mass weight of the wet over the ‘dry’ membrane. The proton conductivity of the membranes was measured in the plane direction at 100 kHz using four-point probe alternating current electrochemical impedance spectroscopy with an electrode system connected to an LCR meter (HIOKI 3522 LCR HiTESTER, Nagano, Japan). For the determination of the conductivity in liquid water, the membrane was equilibrated in H_2_O at 298 and 353 K and placed between two platinum electrodes in air. For the measurement of the conductivity at different hydration levels from the vapour phase and different temperatures, the membrane was placed in a BT-115 Conductivity Cell (Scribner Associates, Southern Pines, North Carolina, USA) equilibrated by a temperature/humidity controller (HUM-1F, Rigaku Co., Tokyo, Japan) where the RH range was 10–80% at the prescribed temperature within the range 298–333 K. The conductivity σ (mS cm^−1^) was calculated from the obtained resistance *R* (Ω), taking into account the membrane dimensions.

For the SANS experiments in this work, the exchange of the guest molecules in the δ-form clathrates between deuterated chloro­form, which was loaded into the composite membrane during the sulfonation process, to D-Tol or H-Tol was achieved by immersing the films in the new solvent for 1 day, followed by drying at 313 K under vacuum for a few hours.

### 2.2. SANS measurements

The microstructural investigation of two s-sPS membranes, one as-cast film (referred to as M1 in the rest of the paper) and one uniaxially deformed film (M2), was conducted under various RH and *T* conditions by SANS on TAIKAN (BL-15). The M2 membrane was loaded with either H-Tol or D-Tol in the δ clathrate for varying the neutron SLD in the crystalline regions of the sample. A *Q* range between 0.007 and 5 Å^−1^ was covered in reciprocal space by using multiple detector banks in the small-angle (SA), medium-angle (MA) and high-angle (HA) regions, as well as a broad wavelength band between 0.8 and 7.8 Å. Data acquisition and reduction for TOF instruments require special care. The TOF-SANS measures three parameters of each neutron arriving at the detector: the coordinates of the impact position on the detector, which give the scattering angle, and the time. In each time channel, the intensity is measured over a *Q* range defined by the minimum and maximum scattering angles and the wavelength range in the time channel (Hjelm, 1988 ▸). The data from a TOF instrument must be mapped into *Q* space, and the strategy used to do this affects the final resolution of the measurement. However, a major advantage of TOF instruments at pulsed sources is that decisions regarding the intensity/resolution trade-offs in *Q* space can be made after the experiment is complete, as discussed in detail by Hjelm (1988 ▸) and Seeger & Hjelm (1991 ▸). Hjelm (1987 ▸, 1988 ▸) has shown that the most efficient TOF data acquisition scheme is achieved by matching the contribution of the instrument geometry with that of the TOF measurement. Since the matching cannot be done simultaneously for all scattering angles in a time channel, the TOF acquisition scheme for most practical work should be such that the time width is chosen to be constant with as small a value as possible, at least smaller than the instrument geometric term of the resolution (Ishikawa *et al.*, 1986 ▸; Hjelm, 1988 ▸). Additionally, it is not the *I*(*t*) (TOF spectrum) that is treated in the data procedure but the converted *I*(λ), because wavelength-dependent corrections are required for the incident spectrum shape, detector efficiency and sample transmission. Because Δλ/λ = *Δt*/*t*, at TAIKAN a constant Δλ/λ = 0.01 acquisition scheme is used. After application of typical wavelength-dependent corrections (Seeger & Hjelm, 1991 ▸), *I*(*Q*, λ) is created by first setting Δλ/λ = 0.01 and then performing circular averaging and integration of *I*(*Q*, λ) to obtain *I*(*Q*) with constant Δ*Q*/*Q* between 0.02 and 0.05, with the required resolution for data analysis at each detector bank. The resolution at TAIKAN is discussed in detail by Takata *et al.* (2015 ▸). The humidity/temperature on the sample was adjusted using a versatile precision dewpoint generator (Micro-Equipment Inc., Tokyo, Japan) that operates on mixing H_2_O and D_2_O vapour flows in various ratios in a controlled way, and a multi-position sample chamber equipped with temperature control (283 to 358 K). The hydration system uses the two-temperature method for providing the film samples with the required RH within the range 10–85% within a temperature range of 293–358 K under the requested neutron contrast conditions (Arima-Osonoi *et al.*, 2020 ▸). The multi-position sample chamber provided a wide angular range for the scattered neutrons, which enabled full use of the extended *Q*-range capabilities at TAIKAN.

For comparison, to emphasize the capability of the extended *Q*-range SANS investigation with adjusted resolution for structural characterization of semi-crystalline systems, a SANS investigation of a uniaxially deformed s-sPS film (M0) with a higher SD and crystallinity was carried out at the pinhole SANS diffractometer KWS-2 (Radulescu *et al.*, 2015 ▸; Houston *et al.*, 2018 ▸) up to *Q*_max_ = 1.0 Å^−1^, which is the highest *Q* value covered at the instrument when neutrons with λ = 2.8 Å are used in combination with a detection distance of *L*_D_ = 1.1 m. This wavelength is provided by a velocity selector inclined with an angle of −10° from the beam axis. Owing to the use of the velocity selector in an inclined configuration, a wavelength distribution of Δλ/λ = 20% is delivered, which, for a parallel configuration to the beam axis, would otherwise be Δλ/λ = 10%. However, for the lowest wavelength λ = 2.8 Å at the highest speed of the selector the wavelength distribution is Δλ/λ = 14%, due to wavelength cut-off provided by the instrument neutron guide (Houston *et al.*, 2018 ▸). Additional measurements at *L*_D_ = 2, 8 and 20 m were carried out with a neutron wavelength λ = 5 Å, the standard working mode at the instrument, with the selector in normal configuration, parallel with the beam axis, hence using Δλ/λ = 10%. The M0 film sample was measured at KWS-2 at different RH and *T* conditions using an Anton Paar humidity chamber (Schiavone *et al.*, 2018 ▸).

Typical SANS corrections were performed at both instruments (Radulescu *et al.*, 2016 ▸; Takata *et al.*, 2015 ▸), while calibration of corrected data in absolute units was done using glassy carbon (at TAIKAN) and Plexiglas (at KWS-2) standards. Data collected from cast films were radially averaged over the entire detector area while data from uniaxially deformed films were averaged over narrow angular sectors (±5°) on meridian and equatorial directions. Fig. S1 (in the supporting information) shows an overlay of the sectors on a scattering image collected at KWS-2.

## 3. Results

**Figure 1:**
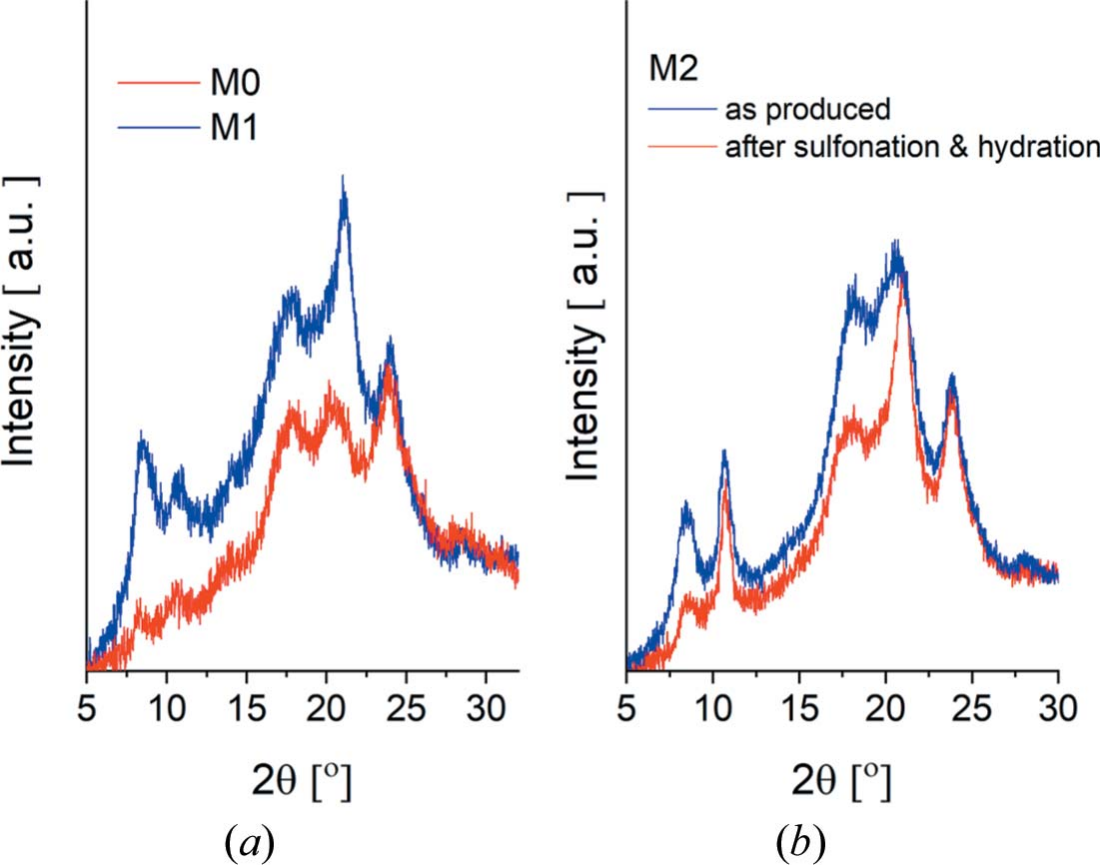
X-ray diffraction patterns from the sulfonated cast M1 and uniaxially deformed M0 films (*a*) and from the uniaxially deformed M2 film as produced and after functionalization (sulfonation), exposure to in-beam hydration and temperature treatment during SANS investigation, followed by the IEC analysis procedure (*b*).

The PGAA characterization of membranes delivered SD values of about 17, 9.0 and 13.5% for the samples M0, M1 and M2, respectively. The guest content determined by TGA varies in the range between 11 and 5 wt%, with the highest value in the M0 sample and the lowest one in the M2 sample. The crystallinity of the film samples as characterized by XRD at a powder diffractometer (Fig. 1 ▸) was about 25, 42 and 38% for the samples M0, M1 and M2, respectively. Knowing the measured IEC and WU parameters, the water content, λ = mol H_2_O/mol SO_3_^−^, could be estimated according to the procedure described by Kusoglu & Weber (2017 ▸). A water content λ of 12.0, 7.5 and 10.5 was obtained for the film samples M0, M1 and M2, respectively, in full hydration (liquid water) state. At RH = 85% the water content in membranes M1 and M2 was about 4.5 and 6.0, respectively. The results from the analysis by PGAA, TGA, XRD (crystallinity), and the water content λ of the three membranes are reported in Table 1 ▸.

As mentioned in the *Introduction* [Sec sec1], one of the most important properties of sPS is its polymorphic behaviour. The trans-planar phases α and β are obtained by melt crystallization processes, while the thermodynamically favoured phase β can also be obtained by solution casting at high temperatures (Rizzo *et al.*, 2002 ▸). Solvent-induced crystallization, either by casting films from solution or by exposure of amorphous films to vapours or by immersion in solvent, leads to the formation of the helical co-crystalline forms δ and ε. The γ form is obtained either by annealing or by heating the clathrate form δ in the temperature range of 383–443 K, which purges the solvent molecules and forms closely packed helical chains in the crystal lattice, or by solution crystallization processes with solvents too large to be trapped as guests of the sPS clathrate phase. Detection of the crystalline phases of sPS is usually performed by XRD, which provides insight into the structural organization and arrangement of host and guest molecules in sPS clathrates on a local scale of a few ångströms. A detailed characterization of typical XRD patterns of unoriented semi-crystalline s-PS samples containing different crystalline forms was reported by Rizzo *et al.* (2002 ▸). The diffraction patterns of all the film samples we studied (Fig. 1 ▸) show two distinct peaks at 2θ ≈ 8 and 10°, indicating the δ form of clathrates with guest molecules, with the two peaks denoting, respectively, the 010 and 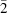 10 reflections of the δ form (Rizzo *et al.*, 2002 ▸). These reflections usually occur when a large number of guest mol­ecules are incorporated in the cavities between the sPS helices (De Rosa *et al.*, 1997 ▸), and represent a pattern used to recognize this crystalline form and the state of guest loading when such experimental data are first examined. As the amount of guest contained in the clathrates decreases, the reflection 2θ ≈ 10° decreases and almost disappears for the depleted clathrate form δ_e_, a behaviour accompanied by an increase in the intensity of the broad feature at 2θ ≈ 14°, which reaches its maximum for the form δ_e_. The observation of the two distinct reflections at 2θ ≈ 8 and 10° and the weak, rather indistinguishable feature at 2θ ≈ 14° indicates that all samples contain clathrate in the δ form with toluene guest molecules. Fig. 1 ▸(*b*) shows the XRD patterns of film sample M2 before and after functionalization by sulfonation, followed by SANS characterization with variable humidity and temperature in the beam and subsequent IEC determination. The difference in the intensity of the reflections is due to the complex treatment of the sample after its preparation according to the protocol described in Section 2.1. Although the crystallinity of the film changed (38% as prepared), it was maintained at a high level (32%) after functionalization and treatment during and after SANS.

The proton conductivity of the membranes depends on the amount of water absorbed, which in turn depends on the SD of the membranes and the RH to which they are exposed. Table 2 ▸ shows the proton conductivities of the film samples M1 and M2 at different temperatures and humidities. The film samples show increasing proton conductivity with increasing RH at room temperature, up to σ = 0.75 and 16.5 mS cm^−1^ for film samples M1 and M2, respectively, when equilibrated in water. Film sample M0 has proton conductivity σ = 36.0 mS cm^−1^ when equilibrated in water at room temperature, showing that the ionic conductivity of such membranes increases with increasing water content λ under these conditions. (Table 1 ▸). Moreover, the s-sPS membranes exhibit comparable ionic conductivity to Nafion under similar hydration conditions at room temperature (Kusoglu & Weber, 2017 ▸).

At a limited humidity (RH < 100%), the ionic conductivity of the membranes decreases with increasing temperature (Table 2 ▸). At RH = 85% and *T* = 303 K, the proton conductivity of the M2 membrane was 0.386 mS cm^−1^, and it decreased with increasing temperature to 0.109 mS cm^−1^ at 323 K and 0.011 mS cm^−1^ at 353 K. The same temperature behaviour of proton conductivity was observed for film sample M1, although the values are lower due to the lower SD of the membrane. In general, all membranes of this type show an Arrhenius behaviour of ionic conductivity, *i.e.* it increases with increasing temperature when equilibrated in water, whereas at a limited humidity (RH < 100%), the membrane conductivity decreases with increasing temperature, regardless of the SD of the membrane, as shown in our previous study (Schiavone *et al.*, 2022 ▸). Thus, the temperature behaviour of ionic conductivity under limited humidity is very different for membranes based on s-sPS compared with Nafion, which shows increasing conductivity with increasing temperature and humidity (Kusoglu & Weber, 2017 ▸).

**Figure 2:**
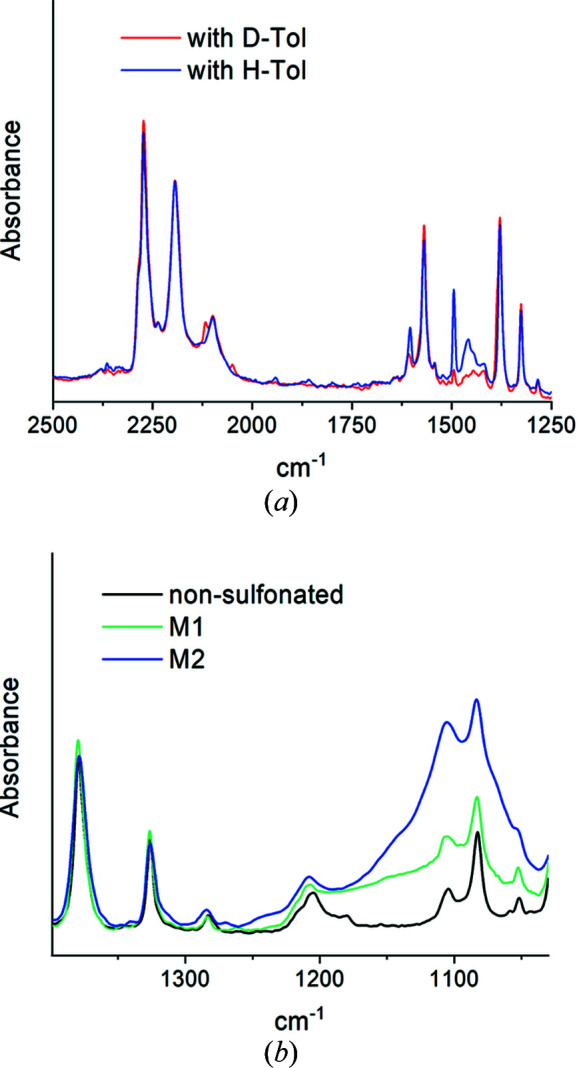
The FTIR spectra from D-Tol and H-Tol loaded deuterated s-sPS film M1 in the spectral region corresponding to the ring modes of the deuterated sPS (*a*) and from films M1 and M2 in the spectral region corresponding to the sulfonic group modes (*b*), in parallel with the non-sulfonated as-prepared M1 film sample.

Fig. 2 ▸(*a*) shows FTIR spectra from M1 loaded with either H-Tol or D-Tol as guest molecules in the crystalline region. Extensive FTIR results on other sPS membranes in either as-prepared or sulfonated states and loaded with either protonated or deuterated guest molecules in the crystalline region were reported elsewhere (Schiavone *et al.*, 2018 ▸, 2019 ▸, 2020 ▸). Typical spectral features of deuterated sPS can be observed in Fig. 2 ▸(*a*), such as the ring stretching and bending modes and backbone C–D and C–D_2_ stretching in the region 2150–2300 cm^−1^ and the C=C stretching at around 1570 cm^−1^. The band at around 1490 cm^−1^ indicates the incorporation of H-Tol in the membrane, and can be used as a marker for the guest-exchange procedure. The region of the symmetric and asymmetric stretching of the SO_3_^−^ group is shown in Fig. 2 ▸(*b*) for the samples M1 and M2 in parallel with the M1 sample before the sulfonation. The stretching vibration ν_as_ (SO_2_) of *R*–SO_3_^−^ compounds generally shows a strong and broad band in the range 1150–1250 cm^−1^, while the in-plane bending vibration of the benzene ring substituted with –SO_3_^−^ and –SO_3_H yields spectral features in the range 1100–1127 cm^−1^ (Zhou *et al.*, 2007 ▸). FTIR complemented the PGAA results and confirmed the successful sulfonation of the membranes.

**Figure 3:**
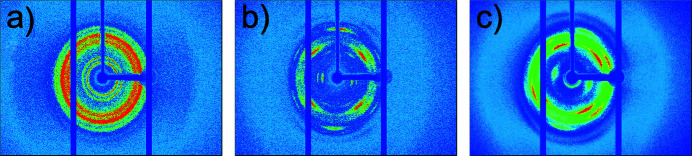
X-ray diffraction patterns from cast (*a*) and uniaxially deformed (*b*), (*c*) films. Patterns (*a*) and (*b*) correspond to sulfonated films while (*c*) corresponds to the sulfonated film in (*b*) after exposure to in-beam hydration and temperature treatment during SANS investigation followed by the IEC analysis procedure.

The membranes M1 and M2 contain δ-form clathrates with toluene guest molecules. The 2D XRD patterns from these samples measured with a single-crystal diffractometer are characterized either by uniform diffraction rings, in the case of the as-cast M1 film sample [Fig. 3 ▸(*a*)], or by diffraction arcs indicating the axial orientation of the crystalline phases, in the case of the uniaxially deformed M2 film sample [Fig. 3 ▸(*b*)]. The XRD samples shown in Figs. 3 ▸(*a*) and 3 ▸(*b*) are from the sulfonated film samples, while the sample shown in Fig. 3 ▸(*c*) is from the sulfonated film sample after being exposed to humidity/temperature variations, treated for the IEC measurement procedure and then dried. Note that the membrane M2 was positioned in the X-ray beam with the stretching axis vertical in the measurement delivering the pattern in Fig. 3 ▸(*b*), while for the last measurement [Fig. 3 ▸(*c*)] the orientation slightly deviated from the vertical positioning.

The orientation of the crystalline phase in the uniaxially deformed film sample after in-beam treatment during SANS characterization and subsequent exposure to IEC measurement conditions is still clearly visible, indicating that the orientation of the crystalline lamellae in the membrane was maintained after all these procedures, although the small-angle reflection signal in Fig. 3 ▸(*c*) appears somewhat blurred. As mentioned earlier, it can be observed from the powder XRD analysis that the degree of crystallization of membrane M2 decreased to some extent after the film sample was exposed to different *T*/RH conditions and chemically treated for the IEC determination procedure. The presence of the double reflection at small angles (about 8 and 10° in 2θ) is indicative of the δ crystalline form with toluene guest mol­ecules (De Rosa *et al.*, 1997 ▸), in which the chains are in the [–(T2G2)2–] helical conformation and the guests are accommodated in the vacancies between the sPS helices.

The SANS patterns from ion-conducting membranes provide information for the analysis of the structures and morphologies that characterize such systems (Gebel & Diat, 2005 ▸). Generally, the *I*(*Q*) from SANS measurements on hydrated membranes shows three features in the representative *Q* regimes: (i) a small-angle upturn at *Q* < 0.01 Å^−1^, representative of the large-scale fractal character of the membrane, (ii) a first broad peak at intermediate 0.01 < *Q* < 0.1 Å^−1^, which is related to the interlamellar spacing in the crystalline domains of the polymer matrix, and (iii) a second peak at *Q* > 0.1 Å^−1^, the ionomer peak that arises from the correlation of ionic clusters, either in the dry or hydrated state. Extended *Q*-range SANS towards higher *Q* values, hence the WANS regime or neutron diffraction, may reveal the scattering pattern of the crystalline reflections [called feature (iv) further on in the paper] due to the oriented crystalline planes in the crystalline lamellae, when proper sample orientation in the beam, neutron contrast and instrumental resolution are provided.

**Figure 4:**
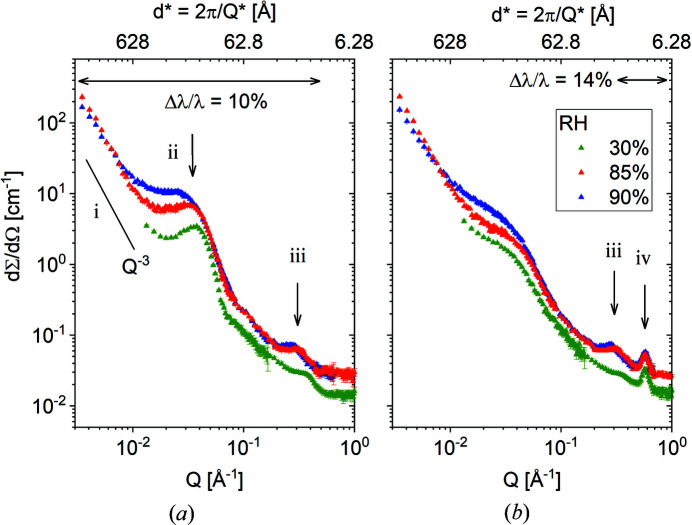
SANS patterns from the sample M0 exposed to different RH states at room temperature, as averaged over the meridional (*a*) and equatorial (*b*) sectors. The scattering features are indicated by roman numbers as follows: (i) the power-law behaviour at low *Q*; (ii) the interlamellar reflection; (iii) the ionomer peak; (iv) the 010 crystalline reflection. Δλ/λ in different *Q* ranges is also indicated.

Fig. 4 ▸ presents the 1D scattering data collected at KWS-2 in pinhole mode from the M0 membrane at room temperature as it was exposed to RH = 30%, RH = 85% and RH = 90%. The data from the uniaxially deformed membrane were averaged over the meridian sectors [panel (*a*)], parallel to the sample stretching direction, and equatorial sectors [panel (*b*)], perpendicular to the stretching axis. The analysed detection sectors are indicated in Fig. S1. The power-law behaviour of the scattering at low *Q* is due to large-scale inhomogeneities (fractal character) in the membrane. The long-range interlamellar correlation peak [scattering feature (ii)] is observable only in the meridional patterns, due to the orientation of the lamellar stacks along the stretching direction, while the ionomer peak [scattering feature (iii)] is observable in both sectors, since it denotes the correlation length between ionic clusters which are randomly distributed within the amorphous regions of the sample. A prominent peak is observed at around *Q* = 0.56 Å^−1^ in the equatorial scattering patterns [scattering feature (iv)], in a position unchanged with the increase in RH. As reported in early work, this is indicative of the 010 crystalline reflection due to correlations between the (010) crystalline planes in the crystalline lamellae when proper neutron contrast is provided (Schiavone *et al.*, 2019 ▸). The Δλ/λ used in different *Q* ranges is also indicated in the figure.

**Figure 5:**
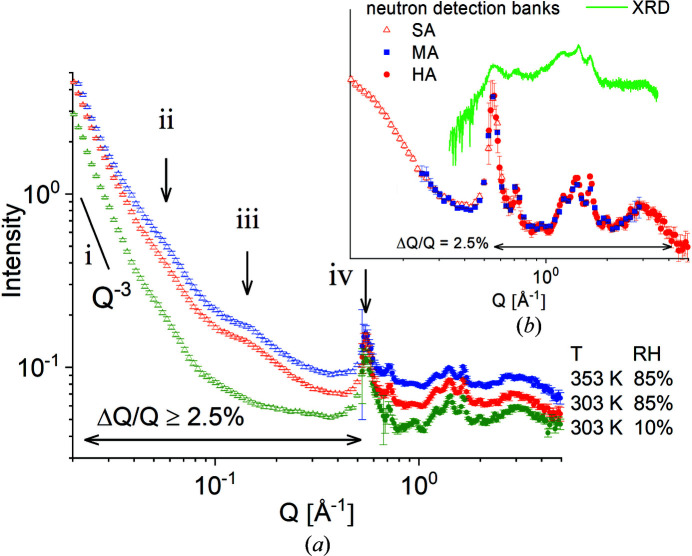
SANS patterns over an extended *Q* range from the sample M1 exposed to different RH states and temperatures (as indicated on the right side of the plot). Data collected on different detector banks in TOF mode at the TAIKAN instrument are depicted as follows: light-coloured symbols – SA, strong-coloured symbols – HA. The scattering features indicated by the roman numbers have the same meaning as in Fig. 3 ▸. The inset shows the SANS data measured at 303 K and RH = 85% over different detection banks in parallel with the XRD data collected at a powder diffractometer. The resolution in different *Q* ranges is also indicated.

Fig. 5 ▸ displays the combined SANS and WANS patterns from the M1 film sample at different temperatures and RH levels as measured over a wide *Q* range at the TOF instrument TAIKAN. The data were obtained with high resolution, which is also indicated on the plot; in the SANS range the resolution decreases with increasing *Q* up to Δ*Q*/*Q* = 2.5%, while in the WANS range the resolution is almost constant, around Δ*Q*/*Q* = 2.5%. Sample M1 is a membrane that is characterized by randomly oriented morphologies. Therefore, the SANS patterns are isotropic and the 1D plots were obtained by radial averaging over the entire detection area. Data measured on the SA and HA detection banks exhibit a fair amount of overlap and are shown in the main plot [Fig. 5 ▸(*a*)], while the inset [Fig. 5 ▸(*b*)] shows data measured at *T *= 303 K and RH = 85% collected with the SA, MA and HA detection banks along with the XRD pattern.

The characteristic scattering features of a semi-crystalline polymer ionomeric membrane are indicated as in Fig. 4 ▸. Dealing with a cast film sample and randomly oriented morphology components, the interlamellar correlation peak [feature (ii)] appears pretty smeared out in the scattering patterns. The ionomer peak [feature (iii)] is observable only in the condition of high hydration for this less sulfonated membrane. On the other hand, the WANS patterns show very prominent peaks, unlike the SANS features. The peak positions measured with WANS and XRD (powder diffraction) are in very good agreement. The peak intensities between neutron and X-ray patterns are different and depend on the scattering contrast characteristic of the two methods. Using H-Tol as guest molecules incorporated in the crystalline region of the deuterated sPS, a high neutron contrast is provided between the crystalline planes and the cavities in the co-crystalline lattice which are filled with guest molecules. Also, we note the very good agreement between data collected with different detection banks, especially between the MA and HA data.

**Figure 6:**
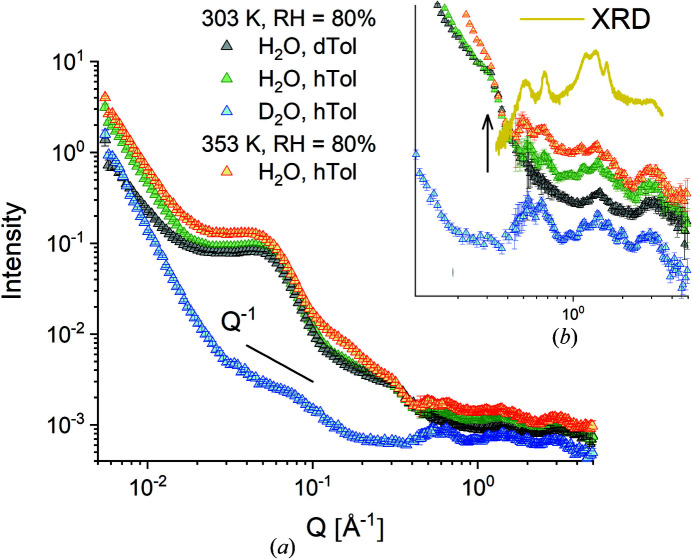
SANS patterns measured in TOF mode at TAIKAN from the sample M2 in different contrast conditions as exposed to different RH/*T* conditions and averaged over the equatorial sectors. The RH and *T *conditions are indicated in the legend. The main plot (*a*) presents the full patterns measured over a wide *Q* range by combining different detection banks, while the inset (*b*) depicts the WANS patterns in parallel with the XRD scattering pattern measured with a powder diffractometer before the SANS characterization. The arrow in the inset (*b*) indicates the ionomer peak.

Figs. 6 ▸(*a*), 6 ▸(*b*) and 7 ▸(*a*), 7 ▸(*b*) report the equatorial and meridional scattering patterns, respectively, from the M2 film sample at RH = 80% as measured at the TOF instrument TAIKAN and collected on the SA and HA detection banks with the sample in different neutron contrast conditions and at different temperatures. The neutron SLD in the functionalized amorphous domains was varied by using either H_2_O or D_2_O hydration water while the neutron SLD in the crystalline region was varied by using either D-Tol or H-Tol as guest molecules in the δ-clathrate phase. Again, the typical scattering features, the interlamellar correlation peak and the crystalline peaks, are easily recognized, while the ionomer peak, although not as clear as in the other samples, can still be seen as a shoulder at about *Q* = 0.3 Å^−1^, as indicated by the vertical arrow in Fig. 6 ▸(*b*). The rich structure of the WANS peaks is observed again, as in the case of the M1 film sample (Fig. 5 ▸), but with differences between the equatorial and meridional scattering patterns, in agreement with the 2D XRD image in Fig. 3 ▸(*b*).

The peak positions agree very well with those in the XRD (powder diffraction) pattern collected in the dry state [Fig. 6 ▸(*b*)]. The intensity of these scattering features varies with the variation of SLD in either the amorphous or the crystalline phase. The ionomer peak [feature (iii)] is no longer visible in the scattering pattern when the hydration is achieved with D_2_O, which is in agreement with the previous observations reported by Schiavone *et al.* (2019 ▸) for other s-sPS membranes. On the other hand, when the membrane is loaded with D-Tol in the clathrate phase, the neutron contrast between the crystalline planes of deuterated sPS helices and the cavities filled with D-Tol in the clathrate phase is reduced and some of the crystalline peaks, such as 010 and 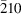 , vanish or are observed with reduced intensity in the WANS region of the scattering patterns compared with the case when the cavities in the δ co-crystals are filled with H-Tol. At high temperature (353 K) and a high hydration level (RH = 80%), although this picture holds, an additional scattering feature appears at intermediate *Q* values around 0.2 Å^−1^, which is observable in both the equatorial and meridional patterns.

## 4. Discussion

The basis of the model interpretation of the scattering patterns recorded from uniaxially deformed s-sPS films in the hydrated state was the assumption that hydration, occurring only in the functionalized regions of the amorphous phase, leads to the formation of hydrated clusters which are randomly distributed in the film sample and thus provide isotropic scattering. Meanwhile the orientation of the crystalline lamellae remains unaffected by water uptake, although water accumulates in the amorphous interlamellar layers too. Thus the scattering from the lamellar stacks appears only in the meridional sectors, mainly as correlation peaks, in addition to the scattering signal from the hydrated clusters. This scenario was confirmed by SANS on very high SD s-sPS membranes hydrated up to 95% RH (Schiavone *et al.*, 2018 ▸). The hydrated clusters, which increase in size and number with increasing RH, define spherical water domains which provide a scattering that could be described by a spherical form factor with polydispersity in size included. Depending on the SD level of the membrane, which defines the density of ion clusters formed as a result of sulfonation, and the crystallinity of the membrane, which defines the volume available for cluster formation, a structure factor combined with the spherical form factor may be required to correctly describe the equatorial SANS profile of correlated or uncorrelated water domains. The meridional SANS profile can be described by a combination of the scattering from the water domains and the scattering from a lamellar stack with a para-crystalline structure factor (Radulescu *et al.*, 2008 ▸), as shown by Schiavone *et al.* (2018 ▸, 2019 ▸).

As observed in Fig. 4 ▸(*b*), the equatorial scattering pattern from the M0 sample presents at intermediate *Q* values, between 0.03 and 0.08 Å^−1^, a smooth Guinier-like profile, which may be assumed as corresponding to a size level that can be described by a spherical form factor. On the other hand, the equatorial scattering pattern of the M2 sample [Fig. 6 ▸(*a*)] shows a steeply declining profile in the same *Q* region, which could indicate a correlation effect that would require a combination of a spherical form factor and a structure factor for a correct description of the scattering data. Therefore, the overall scattering by the hydrated morphology formed in the film samples M0 and M2 was interpreted from the equatorial scattering data using a model that takes into account the superposition of different scattering contributions from morphologies and size levels that are quite well separated on the length scale, as follows: at low *Q*, the scattering from the large-scale inhomogeneities (fractal character) in the membrane, whose size is larger than the length scale detected by the diffractometer, is described by an asymptotic power-law behaviour; at intermediate *Q*, the scattering of spherical water domains, which have either a monomodal or a bimodal size distribution, is described by the spherical form factor *P*(*Q*) for one or two very different domain sizes, with polydispersity in size included; and the ionomer peak is present at high *Q*, which indicates the correlation distance between hydrated ion clusters. The correlation between water domains is accounted for by multiplying the corresponding form factor by a hard-sphere structure factor *S*(*Q*) (Schiavone *et al.*, 2019 ▸). The details and results of the fitting procedure are given in the supporting information, together with the structural and scattering parameters resulting from the model interpretation of the scattering patterns. As can be seen, the membrane M0 data are described by considering only one type of spherical hydrated domain, whereas two types of hydrated domains were considered for the interpretation of the data from the membrane M2, with the larger species being affected by correlation effects. We can interpret this difference by considering the differences in crystallinity of the two membranes. Membrane M0 is characterized by a lower crystallinity, so there is a larger fraction of amorphous regions in the sample than in membrane M2. Therefore, the water domains form and grow in the membrane M0 in a less restricted manner than in the film sample M2, where the higher crystallinity limits the development of the hydrated domains. In addition, the measured conductivity in liquid water is higher for membrane M0 than for the M2 film sample, partly due to a higher degree of functionalization, but presumably mainly due to more extensive growth of the water pathways in the saturated hydrated state. The correlated spherical water domains in the M2 membrane indicate that the hydrated domains tend to cluster in this sample, making their cross-linking throughout the membrane and development into continuous water channels less pronounced than in the case of the M0 membrane. Another peculiarity of the M2 membrane is the presence of a bimodal size distribution of the spherical water domains, which may also be related to its high crystallinity: the water accumulates around the ion clusters and the hydrated domains grow at different rates in the different regions of the functionalized amorphous phase, with increasing RH. Therefore, the scattering from the smaller species of hydrated spherical domains extends to higher *Q* values, making the appearance of the ionomer peak less obvious.

**Figure 7:**
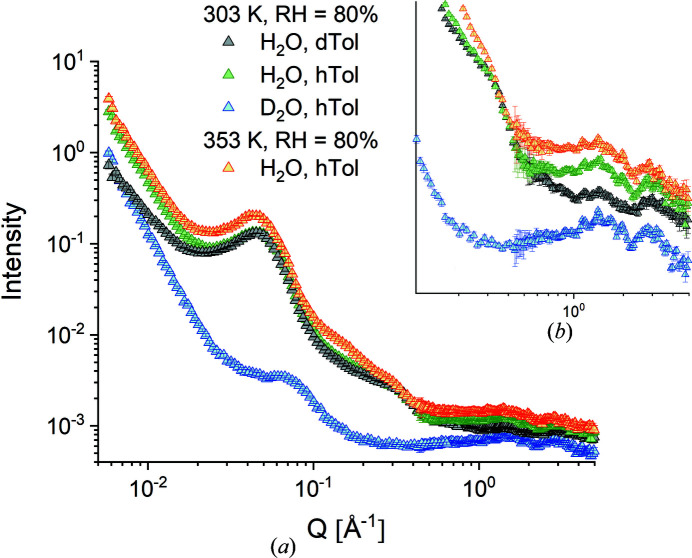
SANS patterns measured in TOF mode at TAIKAN from the sample M2 in different contrast conditions as exposed to different RH/*T* conditions and averaged over the meridional sectors. The RH and *T* conditions are indicated in the legend. The main plot (*a*) presents the full patterns measured over a wide *Q* range by combining different detection banks, while the inset (*b*) depicts the WANS patterns.

The contrast variation in the hydrated regions of the membrane when D_2_O is used instead of H_2_O supports this idea. The scattering intensity is much lower when the deuterated membrane is hydrated with D_2_O, and the scattering features are the result of a subtle but distinct difference in the SLD of the different components of such membranes under these hydration conditions, as discussed by Schiavone *et al.* (2019 ▸). Although the interlamellar correlation peak [Fig. 7 ▸(*a*)] is still visible in the scattering pattern, its intensity is very low and a slight shift in peak position is observed when D_2_O is used instead of H_2_O. At this stage of the study, only a qualitative explanation for this effect can be given. It seems that the first picture of stacks of aligned crystalline lamellae containing sulfonated and hydrated amorphous layers between the lamellae, as we discussed in previous work (Schiavone *et al.*, 2019 ▸, 2020 ▸), is quite crude. We observed that sulfonation swells the interlamellar layers and, although hydration also affects these layers, it does not induce further swelling (Schiavone *et al.*, 2018 ▸, 2019 ▸). We hypothesize that hydration of the interlamellar layers is not homogeneous across the entire layer, and therefore the SLD may vary across the layer thickness, so that hydrated/non-hydrated sublayers may exist. In this case, the neutron contrast in the lamellar stacks will vary in a complicated way depending on whether H_2_O or D_2_O is used. Obviously, for the M2 membrane, the change of hydration conditions from H_2_O [pattern with black symbols in Fig. 7 ▸(*a*)] to D_2_O [pattern with blue symbols in Fig. 7 ▸(*a*)] leads to a change in the correlation effect between the different layers in the stack; in the H_2_O case the distance between the crystalline lamellae is detected as in the dry film sample, while in the D_2_O case a different, smaller spacing can be detected, so that the weaker correlation peak occurs at a higher *Q* value, as observed in Fig. 7 ▸(*a*). However, validation of this assumption requires further detailed studies, where several similar film samples should be investigated simultaneously using contrast-variation SANS, carefully changing the SLD in the samples so that the structure and morphology are not disturbed and only the scattering properties are varied.

The scattering profile of the larger water domains shows a *Q* ^−1^ power-law behaviour under the conditions of D_2_O hydration, as observed in the equatorial scattering pattern. This profile can be explained by the 1D appearance of the hydrated regions formed along groups of elongated s-sPS chains with the same SLD value as the hydration water, in contrast to the surrounding crystalline or non-sulfonated sPS environment, which is characterized by slightly different SLD values. A tentative model interpretation of the scattering pattern in terms of a cylindrical form factor at intermediate *Q* combined with a power-law contribution at low *Q* describes the experimental data quite well for a thickness of the 1D objects of about 30 Å (Fig. S2). No clear scattering feature is observed in the *Q* range of 0.2–0.3 Å^−1^ for this contrast condition. Apparently, the smaller hydrated domains do not produce a sufficiently high neutron contrast in this hydration condition. The ionomer peak, which indicates the correlation distance between ion clusters in the hydrated domains, appears in all scattering patterns when the three membranes are hydrated with H_2_O, superposed to some extent with the scattering from the smaller water domains. As previously reported, the ionomer peak occurs as a consequence of the large difference in neutron SLD between the H_2_O (−0.56 × 10^10^ cm^−2^) and the sulfonated segments of the deuterated sPS (6.34 × 10^10^ cm^−2^). Upon hydration with D_2_O (6.38 × 10^10^ cm^−2^), the neutron contrast between the sulfonated segments of the deuterated sPS and the hydrated environment vanishes, and so does the ionomer peak in the scattering pattern [Figs. 6 ▸(*b*) and 7 ▸(*b*)].

The most important information about the hydrated domains in membrane M2 comes from the comparison of the scattering patterns and fitting parameters at different temperatures. From the results shown in Figs. S3 and S4 and the parameters given in Table S1, it is evident that the scattering contribution of the smaller uncorrelated spherical water domains increases with increasing temperature, while the RH remains constant. At the same time, these water domains shrink in size at high *T*, leading to the conclusion that their number must increase, since a higher ‘forward scattering’ from these scattering objects was obtained at 353 K than at 303 K. This effect is accompanied by a slight decrease in the size and ‘forward scattering’ of the larger water domains, a slight increase in the ‘hard sphere’ volume fraction and the ‘hard sphere’ radius, and also a slight decrease in the correlation distance between ionic clusters. This indicates a decrease in the size of the large water domains and, in general, a decrease in the size of all water domains, bringing the ionic clusters closer together. When this structural information is combined with the observed drastic decrease in the conductivity of this membrane with increasing temperature at limited RH < 100%, it can be concluded that the water domains appear to split and shrink with increasing temperature and lose their interconnection, leading to interrupted water pathways in the membrane and consequent loss of its conductive properties.

The novelty of our current study is the possibility to acquire WANS data simultaneously with SANS characterization of such semi-crystalline polymer films. The WANS data of film samples M1 and M2 agree very well in peak position with the XRD data of the same samples, demonstrating the quality of the recorded WANS data. The observation of the 010 and 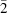 10 reflections in Figs. 5 ▸(*a*) and 5 ▸(*b*) in the *Q* range of 0.5–0.8 Å^−1^ indicates that the sample contains the sPS δ clathrate with H-Tol. Other peaks are clearly observed in the *Q* range 1.0–2.0 Å^−1^, which can be attributed to the combination of reflections due to different (*hkl*) planes, as shown by Rizzo *et al.* (2002 ▸). Both the XRD and WANS scattering patterns shown in Fig. 5 ▸(*b*) are consistent with the XRD scattering patterns of the δ form reported in this paper, demonstrating that good-quality crystallographic data can be obtained with WANS over an extended *Q* range if appropriate resolution is used. Moreover, the combination of SANS and WANS using the same neutron instrument offers the possibility to simultaneously characterize the local crystalline and the large-scale amorphous regions of semi-crystalline polymers, while varying the external stimuli on the sample *in situ*. Similar studies can of course be performed with a combination of small-angle X-ray scattering (SAXS) and wide-angle X-ray scattering (WAXS) on the same X-ray instrument, as demonstrated by Martens *et al.* (2019 ▸) on proton-exchange fuel cells that are transparent to high-energy X-rays.

However, as mentioned in the *Introduction* [Sec sec1], neutrons offer the unique advantage of contrast variation which provides additional structural information to that obtained with X-rays, especially in the case of complex semi-crystalline polymeric materials subjected to variable RH and *T*. Therefore, demonstrating that good-quality WANS data can be collected from such materials is a prerequisite for using the SANS–WANS combination on the same neutron scattering instrument over an extended *Q* range up to several Å^−1^. This approach can be very useful in the structural study of semi-crystalline polymer materials for energy applications such as PEEK [poly(ether ether ketone)], PPEK [poly(phthalazinone ether ketone)], PBI (polybenzimidazole) *etc*., which are characterized by much smaller crystalline unit cells than the sPS one and thus provide crystalline reflections at *Q* values above 1.0 Å^−1^. The clear advantage of using uniaxially deformed film samples and SANS–WANS contrast variation over an extended *Q* range can be seen in the example of the M2 membrane. Again, there is good agreement between the XRD and WANS data in terms of the position of the crystalline reflections, as can be seen in Fig. 6 ▸(*b*), and when comparing the occurrence of the peaks in equatorial and meridional directions with the diffraction pattern observed in Fig. 3 ▸(*b*). For example, the first two crystalline reflections 010 and 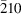 in Fig. 3 ▸(*b*) are only visible in the equatorial direction, which is consistent with the observation of these two peaks in the WANS data analysed in the equatorial sector (the clearest appearance is seen in the pattern shown with blue symbols, which corresponds to the deuterated host lattice with protonated guest molecules). However, the neutrons provide additional information about the crystalline phase of the membrane sample. Thus, loading the deuterated host lattice with deuterated guest molecules [the pattern with the black symbols in Fig. 6 ▸(*b*) and Fig. 7 ▸(*b*)] leads to the disappearance of most reflections. This is because the contrast between the planes of the host polymer and the planes of the guest mol­ecule decreases when both components are used in deuterated form, and the difference in SLD (5.66 × 10^10^ cm^−2^ for D-Tol and 6.34 × 10^10^ cm^−2^ for deuterated crystalline sPS) is much smaller than when a protonated guest is used (SLD = 0.94 × 10^10^ cm^−2^ for H-Tol). Note that the calculated neutron diffraction pattern in the 010 and 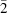 10 reflections of the ethyl­benzene-loaded δ-clathrate form of sPS was obtained on the basis of the alternating layers of polymer and solvent in the (010) direction by Moyses & Spells (1998 ▸). In their calculations, the 010 peak was clearly shown, as was the 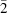 10 peak with less intensity, suggesting that a significant 010 peak could be expected only for the deuterated polymer with protonated guest molecule. Another piece of information provided by the analysis of the WANS patterns is that varying the contrast in the hydro­philic regions of the membrane using D_2_O or H_2_O hydration water or the temperature on the sample, as described above, leaves the WANS intensity unchanged, as can be seen when comparing the blue, green and red WANS patterns in Fig. 6 ▸(*b*). Regardless of the change in background due to the higher incoherent level when the membrane is hydrated with H_2_O, or due to the superposition of the signal from larger hydrated morphologies in the *Q* region at about 0.5–0.6 Å^−1^, the configuration of the crystalline peaks is preserved.

We can clearly state that the validity of the proposed model of spherical hydrated domains formed in functionalized sPS membranes upon water uptake is confirmed by the combined analysis of the results of SANS and conductivity measurements. At low degrees of hydration, water accumulates around the ion clusters in spherical domains. These domains increase in size with increasing RH and eventually interconnect, making the membrane conductive. When the RH is further increased to 100% or the membrane is equilibrated in liquid water, the spherical interconnected water domains evolve into hydrated channels as shown by cryogenic transmission electron microscopy (cryo-TEM) (Schiavone *et al.*, 2018 ▸), and the conductivity continuously increases, as also observed in the current study. In liquid water, membrane conductivity continues to increase with increasing *T* (Schiavone *et al.*, 2022 ▸). However, in limited RH, the conductivity decreases with increasing *T*. Therefore, conductive pathways consisting of interconnected hydrated spherical domains (clusters) that gradually disconnect and shrink with increasing *T* due to morphological changes and rearrangement of water domains can be assumed to be responsible for this observed effect. As can be seen in Figs. 6 ▸(*a*) and 7 ▸(*a*), water desorption does not occur with increasing *T* in these membranes, which are characterized by a low SD level, because the scattering intensity increases at 353 K compared with 303 K. As mentioned above, it is the structural changes and reorganization of the water domains with increasing *T* that cause disconnection of the water domains and disruption of the conduction pathways. This behaviour of proton conductivity under limited RH appears to be a feature of the sulfonated sPS system and was not observed in the Nafion membranes. The two materials, however, are characterized by a different morphology of the hydrated domains. While interconnected spherical water domains were detected in the sPS membranes, cryo-TEM (Allen *et al.*, 2015 ▸) and contrast-variation SANS (Zhao *et al.*, 2021 ▸) revealed a bicontinuous structure of crystalline and amorphous phases in Nafion, a morphology capable of maintaining high conductivity under increasing *T* and limited RH. It can be concluded that sPS membranes can be used in areas where high hydration conditions can be maintained, or in combination with other systems to improve their conductive properties at low hydration while providing high mechanical stability.

Finally, we would like to assess the methodological value of our current study. In terms of structural analysis at the local level, neutrons offer the unique advantage of being able to play with contrast variations in the co-crystals by choosing between protonated or deuterated guest molecules while working with a deuterated host.

**Figure 8:**
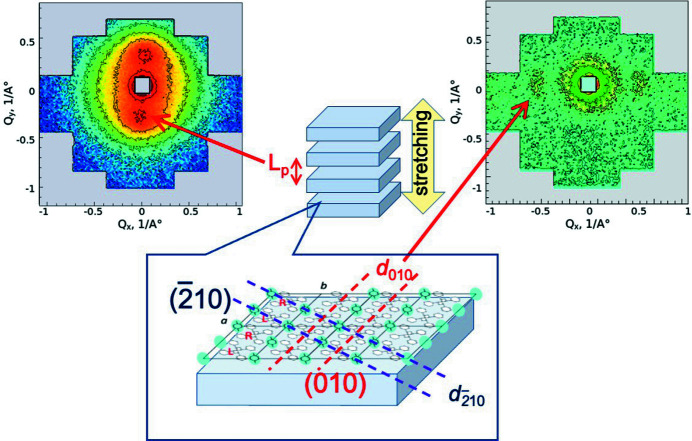
Schematic presentation of the type of scattering patterns produced by different morphology components in a uniaxially deformed semi-crystalline polymer membrane as measured at KWS-2: the observed peaks indicate either the large-scale interlamellar distance between vertically aligned lamellae or the *d*_010_ distance between the (010) crystalline planes aligned horizontally in the crystalline lamellae (*c* axis parallel to the membrane plane). The blue dots represent the guest molecules loaded between the helices of deuterated sPS in the crystalline lamellae.

Fig. 8 ▸ shows schematically which scattering signals result from correlation spacings at different length scales, given the proper neutron contrast and sample orientation. When the film sample is uniaxially deformed, the crystalline lamellae are aligned parallel to each other along the vertical axis. The large corelation distance between the crystalline lamellae leads to peaks in the meridional direction, which are observed with a long-distance detector in a pinhole SANS geometry (in this case *L*_D_ = 8 m at KWS-2). Alignment of crystalline lamellae results in alignment of crystalline planes, with one crystalline plane (*c* plane in Fig. 8 ▸) parallel to the membrane plane (Rizzo *et al.*, 2005 ▸). Correlation spacings between different planes that are perpendicular to the membrane plane result in peaks that lie on the equatorial axis. The low resolution (Δλ/λ = 14%) at KWS-2 only allows observation of correlation distances between the (010) crystal planes when λ = 2.8 Å is used and the detector is set up at *L*_D_ = 1.1 m. Conventional pinhole SANS instruments with a monochromatic beam provided by a velocity selector are equipped with WANS detectors to extend the *Q* range to higher values than those obtained in standard mode, and have the possibility to perform TOF analysis of the scattered neutrons to improve the resolution, but at the expense of intensity (Radulescu *et al.*, 2015 ▸); however, the gain, although definitely very useful, is still limited and inferior to the capabilities of TOF-SANS instruments installed at neutron spallation sources. Although inelastically scattered neutrons (Balacescu *et al.*, 2021 ▸) are a problem for TOF-SANS instruments that may have some impact on the study of solution systems, the broad wavelength band used in these instruments, the static detection geometry, which allows optimal coverage of a very wide angular range, and the tuning of the experimental resolution represent unique advantages in the study of semi-crystalline polymer systems characterized by structural sizes that span a wide length scale, such as the systems discussed in this work.

## 5. Conclusions

An extended *Q*-range (SANS and WANS) experimental approach was used for the structural characterization of functionalized syndiotactic polystyrene membranes under different humidity and temperature conditions. The goal of the current study was twofold. First, we performed a thorough structural characterization of such membranes under different RH/*T* and neutron contrast conditions. Although the mechanical robustness of sPS membranes subjected to hydration and temperature variations has been demonstrated in previous studies and was also shown in the current work by observing the 2D and 1D XRD patterns (collected with either a single-crystal or a powder diffractometer), the ability to collect SANS and WANS scattering patterns from the same membrane during *in situ* hydration and thermal treatment, and thus to simultaneously observe and characterize the large-scale hydrated morphologies and the local crystalline structures, represented the main goal of our structural study. On the other hand, the ability to collect SANS and WANS patterns over an extended *Q* range with an experimental resolution that allows clear observation of the scattering pattern characteristic of a particular crystalline structure, as demonstrated here on the co-crystalline sPS system with guest molecules, may be useful in the analysis of many other semi-crystalline systems characterized by smaller crystalline unit cells than that of sPS and therefore yielding WANS peaks at higher *Q* values. Furthermore, the capability for neutron contrast variation can provide additional structural information on the local crystalline structure and large-scale morphology to that provided by a SAXS–WAXS analysis. This capability paves the way for nanostructure-level evaluation of the mechanical and chemical stability of a material subjected to more severe *in situ* treatment conditions than those described here, such as those used in fuel-cell applications, chemical treatment or composite fabrication.

In the case of sPS membranes, the selective sulfonation of only the amorphous phase makes this material hydro­philic and proton conducting, with similar performance to Nafion. In addition, due to its helical chain conformation in the crystalline phase, sPS can form co-crystals with various small organic molecules, which are incorporated into the vacancies between the polymer helices. The guest molecules can be easily exchanged for other molecules by exposing them to the liquid or vapour of the new guest. These two properties were exploited in the present structural characterization of sulfonated sPS membranes by neutron scattering. The neutron contrast in the crystalline phase was varied by loading either deuterated or protonated toluene as guest molecules into the vacancies between the crystallized chains. This provided information on the crystal structure and, complemented by XRD, showed that the crystallinity of the material was maintained to a large extent during *in situ* hydration and thermal treatment of the sample. Neutron contrast was varied in the amorphous functionalized phase with H_2_O or D_2_O, which provided detailed information about the hydrated domains and conductivity pathways. The combination of structural and conductivity measurements provided a clear understanding of the observed anomalous behaviour of the conductivity of this type of membrane under increasing temperature and limited humidity. The interconnected water clusters in the sPS membranes are a morphology that appears to be responsible for this behaviour, in contrast to the Nafion membranes, which are characterized by a bicontinuous structure of crystalline and amorphous phases that facilitates the development of conductive pathways which are maintained at high temperatures and low humidity.

Finally, SANS and WANS were combined on the same beamline using two types of instruments: a pinhole SANS with a limited WANS option installed at a research reactor, and a ‘small- and wide-angle’ TOF instrument installed at a spallation source. The results obtained have shown the limitations of the pinhole SANS method in terms of resolution and *Q* range when we push the instrument settings towards WANS capability, although useful information can still be obtained about crystallinity in semi-crystalline systems characterized by a large crystalline unit cell, yielding crystalline peaks at about *Q* = 1.0 Å^−1^. On the other hand, TOF-SANS and TOF-WANS on the same instrument at a spallation source are a very suitable approach for the complete structural characterization of such complex morphologies that exhibit multiple size levels spanning a broad length scale from ångströms to hundreds of nanometres due to the broad wavelength band, the fixed detection geometry, which provides the possibility to cover a large angular range, and the tunable resolution that can achieve Δ*Q*/*Q* = 2.5%.

We emphasize here that the combination of SANS–WANS with the possibility of simultaneous acquisition of scattering signals from the local crystalline structure and large-scale morphology has a broad range of applications; this is due to the high versatility of neutron scattering and the possibility to selectively vary the contrast in both crystalline and amorphous phases, and the neutrons’ high penetration power when using complex auxiliary equipment (fuel cells, chemical reaction chambers *etc*.). This can be used to study complex materials with *in situ* variation of conditions relevant to the application.

## Supplementary Material

Fitting of the experimental SANS data and graphical depiction of the detection sectors used for the data analysis on equatorial and meridional directions. DOI: 10.1107/S1600576723005496/jl5067sup1.pdf

## Figures and Tables

**Table 1:** Properties of the s-sPS films after sulfonation and hydration determined by (*a*) PGAA, (*b*) powder XRD, (*c*) TGA, and (*d*) IEC and WU measurements

Sample	SD^*a*^	Crystallinity^*b*^	Guest amount in crystalline phase^*c*^	λ^*d*^
M0	17.0%	25%	11 wt%	12.5 (liquid water)
M1	9.0%	42%	9.5 wt%	7.5 (liquid water)
				4.5 (RH = 85%)
M2	13.5%	38%	5 wt%	10.5 (liquid water)
				6.0 (RH = 85%)

**Table 2:** Proton conductivity of M1 and M2 membranes under different humidity and temperature conditions

RH (%)	Temperature (K)	σ M1 (mS cm^−1^)	σ M2 (mS cm^−1^)
50	303	0.0025	0.038
70	303	0.0046	0.127
85	303	0.0154	0.386
85	323	0.0043	0.109
85	353	0.0021	0.011
Liquid water	303	0.75	16.5
