# Supplementary material for: Extended Q-range small-angle neutron scattering to understand the morphology of proton-exchange membranes: the case of the functionalized syndiotactic-polystyrene model system
Source: J Appl Crystallogr. 2023 Jul 25;56(Pt 4):947–60. doi: 10.1107/S1600576723005496 (PMC10405591; doi:10.1107/S1600576723005496)
Supplement: Supplementary file 1 [file j-56-00947-sup1.pdf]

JOURNAL OF APPLIED CRYSTALLOGRAPHY

Volume 56 (2023)

Supporting information for article:

Extended Q-range small-angle neutron scattering to understand the morphology of proton-exchange membranes: the case of the functionalized syndiotactic-polystyrene model system

Maria-Maddalena Schiavone, David Hermann Lamparelli, Christophe Daniel, Manuchar Golla, Yue Zhao, Hiroki Iwase, Hiroshi Arima-Osonoi, Shin-ichi Takata, Laszlo Szentmiklosi, Boglarka Maroti, Jürgen Allgaier and Aurel Radulescu

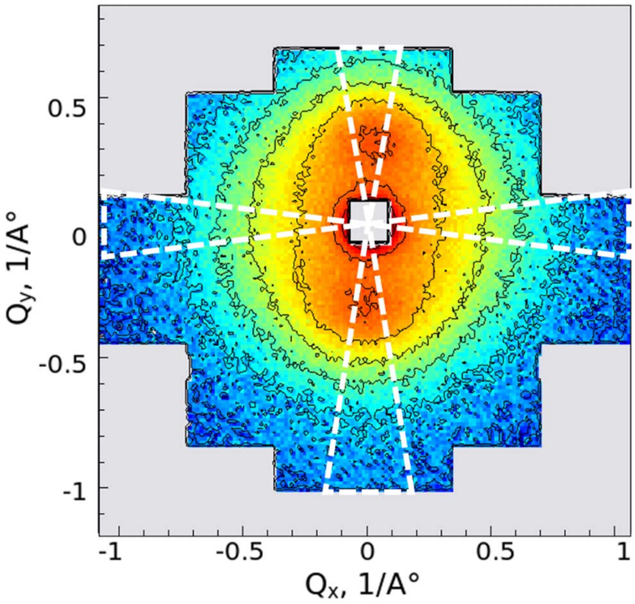

Figure S1 Example of a two-dimensional scattering pattern collected at KWS-2 from a uniaxially deformed s-sPS film sample, and illustration of the angular sectors of ± 5° with white broken line over which the data were analysed on equatorial and meridional sectors.

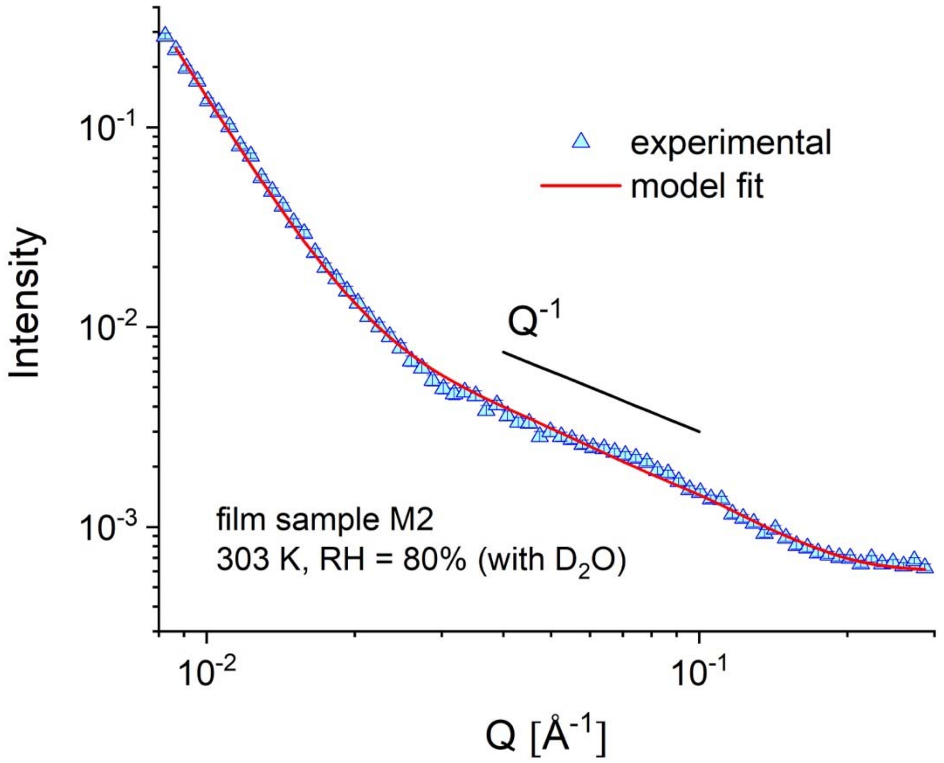

Figure S2 Experimental scattering pattern averaged over the equatorial sectors (symbols) as it was collected at TAIKAN on the small-angle detectors bank from the film sample M2 at T = 303 K and RH = 80%, hydrated with D_2O. Based on the Q^{-1} behaviour of the scattering at intermediate Q, the model interpretation was done in terms of the cylindrical form factor (Radulescu et al., 2008) combined with a power-law contribution for the upturn observed at low Q.

The experimental data averaged over the equatorial sectors from the film sample M0 measured at KWS-2 at 303 K and RH = 90% and from the membrane M2 measured at TAIKAN "small and wide-angle diffractometer" at two temperatures, 303 K and 353 K, and at RH = 80%, were interpreted according to the structural model that considers a superposition of different scattering contributions from morphologies and size levels which are rather well separated on the length scale. At low Q, the scattering from the large-scale inhomogeneities (fractal character) in the membrane with sizes larger than the length scale covered by the diffractometer is described by an asymptotic power-law behaviour; at intermediate Q, the scattering of spherical water domains, which have either a monomodal or a bimodal size distribution, is described by the spherical form factor P(Q) for one or two very different domain sizes, with polydispersity in size included; and at high Q, the ionomer peak, which indicates the correlation distance between hydrated ion clusters. The correlation between water

domains is accounted for by multiplying the corresponding form factor by a hard-sphere structure factor S(Q). Thus, the model can be analytically expressed as:

$$I(\mathrm{Q}) = P_f Q^{-\alpha} + I_0^{sph1}\, P^{sph1}(\mathrm{Q})\, S^{sph1}(\mathrm{Q}) + \ I_0^{sph2}\, P^{sph2}(\mathrm{Q}) + \ \mathrm{G}(\mathrm{Q}) + \mathrm{Bckgd}$$

here P_{f} is the pre-factor of the power-law with the exponent α, I_0^{sph1} is the "forward scattering" of the ensemble of larger size spherical domains of radius R^{sph1}, P^{sph1}(Q) is the spherical form factor of the spherical domains of radius R^{sph1}, S^{sph1}(Q) is the hard-sphere structure factor arising from the intraparticle interference between the spherical domains of radius R^{sph1}, and which depends on the "hard sphere" radius $\mathrm{R_{HS}}$ of the interaction potential and the volume fraction of hard spheres η_{HS}, I_0^{sph2} is the "forward scattering" of the ensemble of smaller size spherical domains of radius R^{sph2}, P^{sph2}(Q) is the spherical form factor of the spherical domains of radius R^{sph2}, G(Q) is the gaussian function describing the ionomer peak, with the amplitude (A_{ion}), width (w_{ion}) and peak position (xc_{ion}) as parameters, and Bckgd represents a constant background line. A detailed description of the spherical form factor and hard-sphere structure factor is reported by Schiavone et al. (2019). Thus, the model contains 11 parameters, which were let free during the fitting procedure, and one parameter, the power-law exponent, which was fixed as determined by the linear fitting of the very low Q data. The global model fit of the data is shown in Fig. S1 and S2 as red curve, while different contributions from the components of the complex morphology are depicted by the curves with different other colours. The fitting parameters are reported in Table S1 (for the ionomer peak, only the peak position is reported). The "forward scattering" $\mathrm{I_0}$ depends on the squared volume of the scattering object, multiplied by the number density of the scattering objects ($\mathrm{N/V_{sample}}$, with N – the number of scattering objects, and $\mathrm{V_{sample}}$ – the sample volume) and the contrast squared and it is usually expressed in $\mathrm{cm^{-1}}$.

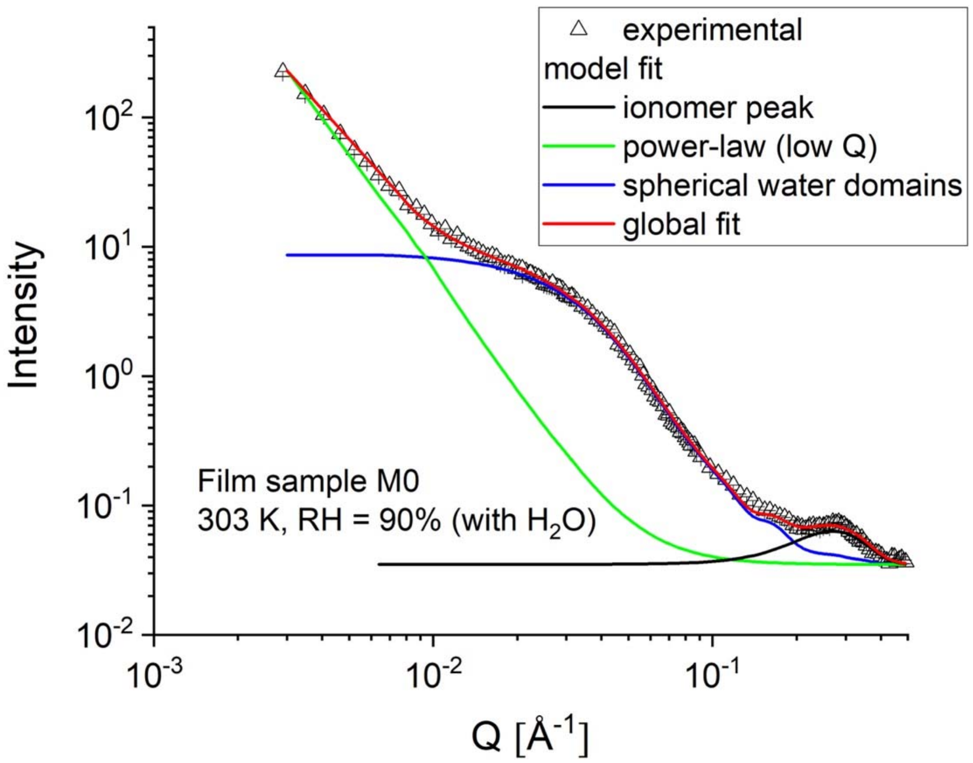

Figure S3 Experimental scattering pattern averaged over the equatorial sectors (symbols) as it was collected at KWS-2 from the film sample M0 at T = 303 K and RH = 90%, hydrated with H_2O and the model interpretation (lines) based on the combination of spherical water domains (blue), ionomer correlation peak (black) and the low-Q power-law behaviour due large-scale inhomogeneities (fractal character) in the membrane (green). The global fit of the experimental data representing the superposition of these contributions is represented by the red line. Parameters of the fitting procedure are reported in Table S1.

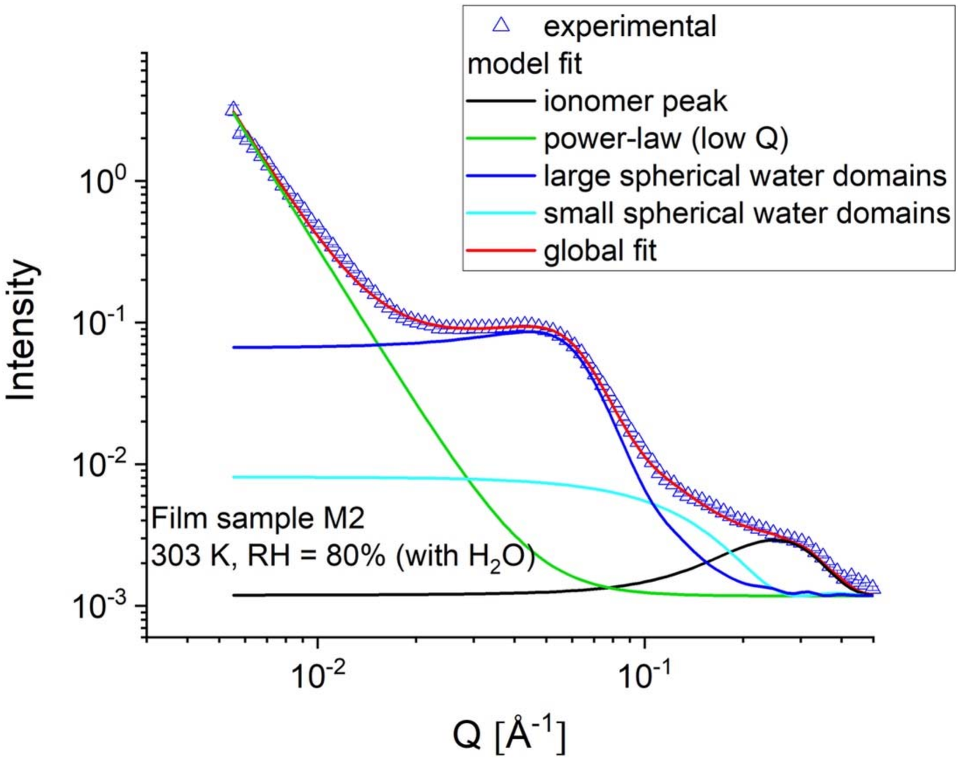

Figure S4 Experimental scattering pattern averaged over the equatorial sectors (symbols) as it was collected at TAIKAN on the small-angle detectors bank from the film sample M2 at T = 303 K and RH = 80%, hydrated with H_2O and the model interpretation (lines) based on the combination of spherical water domains of different sizes (dark and light blue), ionomer correlation peak (black) and the low-Q power-law behaviour due large-scale inhomogeneities (fractal character) in the membrane (green). The global fit of the experimental data representing the superposition of these contributions is represented by the red line. Parameters of the fitting procedure are reported in Table S1.

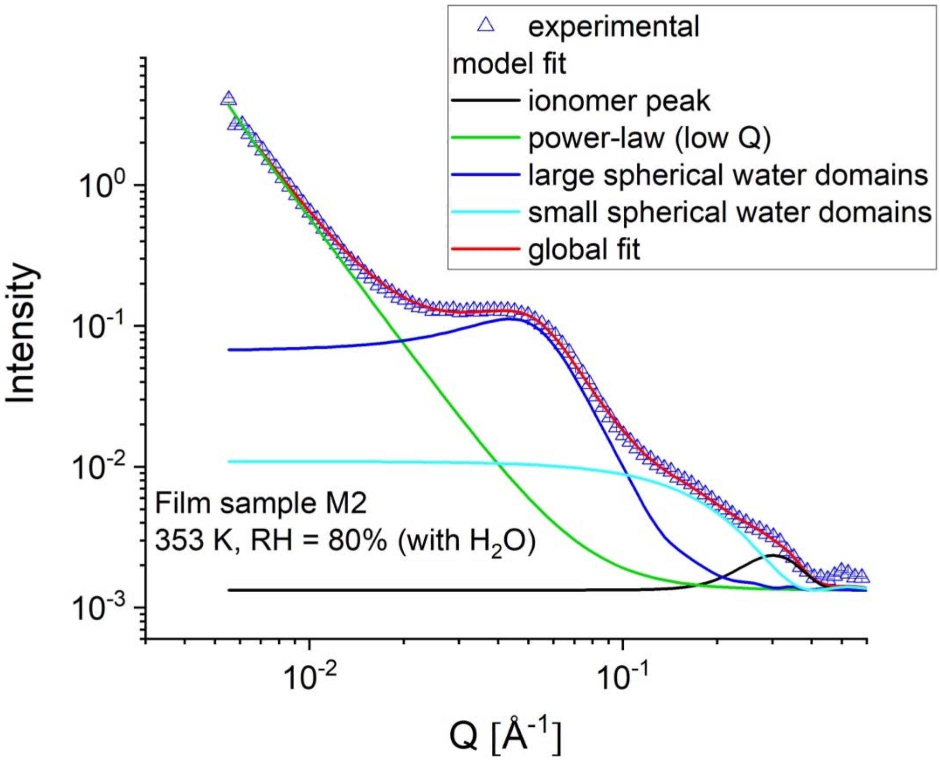

Figure S5 Experimental scattering pattern averaged over the equatorial sectors (symbols) as it was collected at TAIKAN on the small-angle detectors bank from the film sample M2 at T = 353 K and RH = 80%, hydrates with H_2O. The model interpretation and the meaning of the coloured curves is like in Fig. S1. Parameters of the fitting procedure are reported in Table S1.

Table S1 Fitting parameters delivered by the model interpretation of the experimental data in Fig. S3, S4 and S5.

experimental condition / parameter	Membrane M0<br>T = 303 K, RH = 90%	Membrane M2<br>T = 303 K, RH = 80%	Membrane M2<br>T = 353 K, RH = 80%
I_0^{sph1} [cm^{-1}]	9.0 ± 0.3	0.222 ± 0.006	0.214 ± 0.002
R^{sph1} [Å]	65.62 ± 0.84	43.09 ± 0.36	39.94 ± 0.15
η^{sph1}	-	0.1593 ± 0.0032	0.1636 ± 0.0017
R_s^{sph1} [Å]	-	47.86 ± 0.38	55.44 ± 0.27
I_0^{sph2} [cm^{-1}]	-	0.0067 ± 0.0004	0.0096 ± 0.0002
R^{sph2} [Å]	-	15.10 ± 1.3	10.90 ± 0.17
P_f [cm^{-1} Å$^{-\alpha}$]	5.22E-6 ± 2.31E-8	1.4287E-8 ± 9.67E-11	5.88E-7 ± 2.36E-9
α	3.0	3.7	3.0
xc_{ion} [Å]	0.271 ± 0.006	0.248 ± 0.015	0.303 ± 0.003
Background [cm^{-1}]	0.0035 ± 0.0002	0.00118 ± 0.000009	0.00135 ± 0.000009
